# Neuropsychological testing and biomarkers in the management of brain metastases

**DOI:** 10.1186/1748-717X-3-26

**Published:** 2008-09-17

**Authors:** Andrew Baschnagel, Pamela L Wolters, Kevin Camphausen

**Affiliations:** 1 Radiation Oncology Branch, National Cancer Institute, National Institutes of Health, 9000 Rockville Pike, Building 10-CRC, Room B2-3561, Bethesda, Maryland, 20892, USA; 2 Medical Illness Counseling Center and National Cancer Institute, National Institutes of Health, Bethesda, USA

## Abstract

Prognosis for patients with brain metastasis remains poor. Whole brain radiation therapy is the conventional treatment option; it can improve neurological symptoms, prevent and improve tumor associated neurocognitive decline, and prevents death from neurologic causes. In addition to whole brain radiation therapy, stereotactic radiosurgery, neurosurgery and chemotherapy also are used in the management of brain metastases. Radiosensitizers are now currently being investigated as potential treatment options. All of these treatment modalities carry a risk of central nervous system (CNS) toxicity that can lead to neurocognitive impairment in long term survivors. Neuropsychological testing and biomarkers are potential ways of measuring and better understanding CNS toxicity. These tools may help optimize current therapies and develop new treatments for these patients. This article will review the current management of brain metastases, summarize the data on the CNS effects associated with brain metastases and whole brain radiation therapy in these patients, discuss the use of neuropsychological tests as outcome measures in clinical trials evaluating treatments for brain metastases, and give an overview of the potential of biomarker development in brain metastases research.

## Introduction

Brain metastases, the most common intracranial tumor occurring in approximately 10–30% of adult cancer patients and 6–10% of children with cancer, are a major cause of morbidity and mortality [1]. The majority of these tumors metastasize from lung carcinoma, breast carcinoma and melanoma. Patients often present with headaches, nausea and/or vomiting and seizures. Many patients also suffer from some form of neurological and/or neurocognitive impairment which can cause emotional difficulties and affect quality of life. The prognosis for these patients is poor and without therapeutic intervention the natural course is one of progressive neurological deterioration with a median survival time of one month [2]. Patients treated with whole brain radiation therapy (WBRT) have a median survival of 3–6 months [2-5]. The addition of WBRT can relieve neurologic symptoms and prevent death from neurological causes [6].

The best predictor of survival is the Radiation Therapy Oncology Group (RTOG) recursive partitioning analysis (RPA) (Table 1). It divides patients treated with WBRT into three survival classes based on the status of primary tumor control, presence of extracranial metastases, Karnofsky Performance Status (KPS) and age [7]. It has been shown to retain its prognostic value in patients receiving stereotactic radiosurgery (SRS) along with WBRT [8] and when stratifying for different histologies [9,10].

**Table 1:** RTOG RPA classification for brain metastases and associated survival by class in patients treated with WBRT

**Class**	**Patient characteristics**	**Median survival (months)**
I	KPS ≥ 70	7.1
	Age < 65 years	
	Controlled primary tumor	
	No extracranial metastases	

II	KPS ≥ 70	4.2
	One of the following:	
	Age ≥ 65	
	Uncontrolled or synchronous primary disease	
	Extracranial metastases	

III	KPS < 70	2.3

*Abbreviations: *RTOG = Radiation Therapy Oncology Group; RPA = recursive partitioning analysis; KPS = Karnofsky performance status.

Recently a new prognostic index, called the Graded Prognostic Assessment (GPA) has been developed (Table 2) [11]. The GPA uses the sum of scores from four factors: age, KPS, number of CNS metastases, and extracranial disease status. This new index was designed to guide treatment choice, rather than reflect treatment results. It is semi-quantitative, uses the most current data from randomized trials, and has been shown to be as prognostic as the RPA.

**Table 2:** Graded prognostic assessment

	**Score**		
	
	**0**	**0.5**	**1.0**
Age	> 60	50–59	< 50
KPS	< 70	70–80	90–100
No. of CNS metastases	> 3	2–3	1
Extracranial metastases	Present	--	None

**GPA Score**	**Median Survival (months)**		

0 – 1	2.6		
1.5 – 2.5	3.8		
3	6.9		
3.5 – 4	11		

*Abbreviations: *KPS = Karnofsky Performance Status; CNS = central nervous system.

Methods to increase the efficacy of treatment but limit CNS toxicity are currently being investigated. To measure the effectiveness of these emerging treatment modalities various tools will need to be incorporated into clinical trials. Neuropsychological testing and biomarkers are two such useful tools that will assist in optimizing radiation delivery methods and in evaluating agents that modify the effects of radiation. Biomarkers and neuropsychological testing also may aid in making earlier diagnoses, monitoring disease progression, and determining prognosis. This review will briefly summarize the current treatment options available for brain metastases and will review the literature on neuropsychological outcome measures and biomarkers in this patient population.

## Treatment options

Conventional treatment options for brain metastases include whole brain radiation therapy (WBRT), neurosurgery, and stereotactic radiosurgery (SRS), or a combination of the three. Corticosteroids can be used to control peritumoral edema and alleviate neurological symptoms [12]. Chemotherapy traditionally has had a limited role and radiosensitizers are currently being investigated.

### Whole brain radiation therapy

WBRT is considered the standard treatment option for patients who present with multiple brain metastases. It results in a median survival of 3–6 months [2-5], reduces the recurrence rate of metastases, and prevents death from neurological causes [6]. By controlling and improving neurological symptoms, it improves quality of life in 75 to 85% of patients [4]. In addition, WBRT is used in patients with metastases that impinge on important brain structures or are too numerous for either surgery or SRS to be effective. WBRT is used in conjunction with surgery and SRS and its combination has been shown to improve local control [13]. WBRT is effective and, unlike surgery and SRS, it treats both gross and microscopic disease. Table 3 lists the randomized trials that have been performed to determine doses and fractionation schedules of radiation for patients with brain metastases [4,14-20]. The results from these studies showed that the differences in dose, timing, and fractionation do not have a statistically significant difference in median survival. Currently the most common radiation dose in the United States for brain metastases is 30 Gy in ten 3 Gy fractions over two weeks.

**Table 3:** Dose fractionation schedules of randomized trials of WBRT alone

**Study (ref)**	**Year**	***n***	**(Gy)/number of fractions**	**Median Survival (months)**
Harwood et al [14]	1977	101	30/10 vs 10/1	4.0–4.3
Kurtz et al [15]	1981	255	30/10 vs 50/20	3.9–4.2
Borgelt et al [4]	1980	138	10/1 vs 30/10 vs 40/20	4.2–4.8
Borgelt et al [16]	1981	64	12/2 vs 20/5	2.8–3.0
Chatani et al [17]	1986	70	30/10 vs 50/20	3.0–4.0
Haie-Meder et al [18]	1993	216	18/3 vs 36/6 or 43/13	4.2–5.3
Chatani et al [19]	1994	72	30/10 vs 50/20 or 20/5	2.4–4.3
Murray et al [20]	1997	445	54.4/34 vs 30/10	4.5

Survival differences between treatment arms were not significantly different in any study. Adapted from Shaw et al. [30]

Reprinted with permission from the American Society of Clinical Oncology.

### Surgery

Surgical resection is used as a treatment option for patients with a favorable prognosis, surgically assessable metastases and who have minimal extracranial disease [21]. In patients with tumor(s) elsewhere in the body under control, the resection of one or more closely situated metastases can increase survival significantly. Four randomized trials that have been completed to address the role of surgical resection of brain metastases are summarized in Table 4. Three of the trials demonstrated that combining surgery and WBRT for patients with a single metastasis significantly extends survival and improves quality of life when compared to WBRT alone [22-24]. One of the randomized trials failed to show an increase in survival or a benefit in quality of life [25]. However, in this study the patients had lower KPS and a higher incidence of extracranial disease which may have affected the outcome. Overall these results support the position that surgical treatment should be utilized in patients with limited extracranial disease and in those patients with good performance status.

**Table 4:** WBRT vs surgery plus WBRT in randomized trials

**Study (ref)**	**Year**	**Treatment**	**(Gy)/number of fractions**	**n**	**Median survival (mo)**	**P Value**
Patchell et al [22]	1990	Biopsy + WBRT	36 Gy/12	23	3.4	< 0.01
		S + WBRT		25	9.2	
Vecht et al [23]	1993	WBRT	40Gy/10	31	6	0.04
		S + WBRT		32	10	
Noordijk et al [24]	1994	WBRT	40Gy/10	34	6	0.04
		S + WBRT		32	10	
Mintz et al [25]	1996	WBRT	30Gy/10	43	6.3	0.24
		S + WBRT		41	5.6	

*Abbreviatio*n: S = Surgery

### Stereotactic radiosurgery

SRS is an alternative to neurosurgery, in which multiple convergent beams of high energy x-rays, gamma rays, or protons are delivered to a discrete radiographically defined treatment volume. SRS can be used to treat single lesions or multiple lesions (usually up to 3) and can be used to treat deep-seated surgically inaccessible lesions. It has been shown in several large retrospective analyses to be equivalent to surgery [8,26]. Results from one randomized trial and several retrospective studies have shown that when SRS is used after WBRT there is a survival benefit as well as stabilization or improvement in KPS [8,27].

There is no clear consensus on the survival advantage of using SRS followed by adjunct WBRT. A randomized trial by Aoyama et al [28], comparing SRS alone to WBRT plus SRS, did not demonstrate a survival difference in patients with 1 to 4 brain metastases. In this study intracranial relapse occurred more frequently in those who did not receive WBRT [28]. In a phase II trial looking at patients treated with SRS for renal cell carcinoma, melanoma, or sarcoma found that there was a high degree of failures within the brain (approximately 50% of patients by 6 months) with the omission of WBRT [29].

The role of WBRT after SRS remains unclear. Some investigators advocate the omission of WBRT after SRS because SRS has excellent local tumor control for single metastasis and withholding WBRT will spare the patient from the neurocognitive deficits associated with WBRT. Others argue that many patients initially treated with SRS either have micrometastases or will develop recurrent brain metastasis and thus should receive WBRT for local and distant tumor control.

### Radiosensitizers and WBRT

Radiosensitizers are chemicals or biological agents that increase the lethal effects of radiation on the tumor without causing additional damage to normal tissue. Efaproxiral (RSR13) is one example of a radiosensitizer that has shown some promise [30]. It is an allosteric modifier of hemoglobin that works by decreasing the binding affinity of hemoglobin to oxygen thus permitting greater oxygenation of hypoxic tumor cells and enhancing the effect of radiation. In addition to this example, other agents have been investigated in clinical trials (Table 5) [30-37]. Overall these studies have produced mixed results, some have shown a slight survival benefit, while the majority of studies have not shown a difference in survival. These results have not been strong enough to bring any of these agents into routine clinical care. At this time there are several clinical trials underway involving other potential radiosensitizers .

**Table 5:** Trials of WBRT plus radiation sensitizers for brain metastases

**Study (ref)**	**Year**	***n***	**Radioenhancer**	**(Gy)/number of fractions**	**Median Survival (months) WBRT + RS vs WBRT**
Eyre et al. [31]	1984	111	metronidazole	30/10	3.0 vs 3.5
DeAngelis et al. [32]	1989	58	lonidamine	30/10	3.9 vs 5.4
Komarnicky et al. [33]	1991	779	misonidazole	30/6-10	3.9
Phillips et al. [34]	1995	72	BUdR	37.5/15	4.3 vs 6.1
Mehta et al. [35]	2003	401	motexafin gadolinium	30/20	5.2 vs 4.9
Shaw et al. [30]	2003	57	efaproxiral	30/10	7.3 vs 3.4
Suh et al. [36]	2006	515	efaproxiral	30/10	5.4 vs 4.4
Knisely et al. [37]	2008	183	thalidomide	37.5/15	3.9 vs 3.9

*Abbreviations: *RS = Radiation Sensitizer, BUdR = bromodeoxyuridine

### Chemotherapy for brain metastases

The role of conventional chemotherapy has traditionally been limited by the presence of the blood brain barrier and by the potential resistance to chemotherapeutic agents. Conventional chemotherapeutic agents include topotecan, cisplatin, paclitaxel and temozolomide. Temozolomide, a second-generation alkylating agent, has 100% bioavailability and readily crosses the blood-brain barrier. Phase II results show that temozolomide is well tolerated and gives an improvement in response rate [38]. Preclinical data has also shown that temozolomide could be combined with radiation to enhance its effect [39]. Agents that are being currently investigated include gefitinib, lapatinib, valproic acid and thalidomide . Future success of chemotherapy will hinge on the development of new agents that have improved penetration into CNS.

## CNS effects of radiation therapy for brain metastases

WBRT, the standard of care for brain metastases, decreases the tumor burden, which delays neurocognitive decline and maintains quality of life. However, WBRT also can cause brain injury and neurologic complications. There is risk of dementia in long term survivors of brain metastases treated with WBRT [40,41], which is thought to be dependent on the total dose of radiation, the size of the irradiated field, and the fraction size. Understanding and measuring the neurotoxicity associated with WBRT as well as SRS is important for evaluating different treatment regimens beyond the effects on survival and time to disease progression.

### Pathophysiology of radiation induced CNS toxicity

Radiation predominantly causes vascular endothelial damage and demyelination of white matter leading to white matter necrosis [42]. Clinically, radiation injury of the brain can be divided into three categories: acute, subacute and late. Acute effects occur within the first few weeks of radiation treatment and are likely caused by cerebral edema and disruption of the blood brain barrier. Symptoms include drowsiness, headache, nausea and vomiting. Subacute encephalopathy occurs at one to six months after the completion of radiation and its mechanism of damage is believed to be due to diffuse demyelination. Symptoms, which resolve in several months, include headache, somnolence, fatigability, and a transient impairment in cognitive functioning. Late effects are seen six months after radiation and are usually due to damage of the white matter tracts caused by injury to vascular endothelial cells, axonal demyelination, and coagulation necrosis. These late effects usually cause permanent and progressive memory loss and can lead to severe dementia [43].

The incidence of radiation induced dementia is not well studied. The most commonly cited study is from a retrospective review of 47 patients who survived more than one year treated with WBRT [41]. Five (11%) of those patients were reported to develop severe radiation-induced dementia at one year. However, four of these five patients were treated with high radiation fractions (5 or 6 Gy) that are not routinely used. Another study by the same authors reports an incidences of 1.9 to 5.1%, but once again this retrospective review included patients treated with unconventional fractions (4 – 5 Gy) [40]. Contrast enhancing CT findings in these patients reveal cortical atrophy and hypodense white matter. Autopsies on patients with severe radiation induced dementia reveal diffuse chronic edema of hemispheric white matter in the absence of tumor recurrence [40].

The pathophysiology of late radiation injury is a complex process involving damage to oligodendrocytes, endothelial cells, neurons, microglia and astrocytes and the depletion of stem and progenitor cells. It also is a dynamic process that involves recovery/repair responses with release of various cytokines and the involvement of secondary reactive processes that result in persistent oxidative stress [42].

Vascular damage leading to ischemia and consequently white matter necrosis is thought to be a major mechanism for late delayed neurocognitive impairment caused by WBRT. This mechanism is supported by animal experiments designed specifically to study the long-term cognitive effects of rats treated with whole brain radiation. Using this model, investigators found that loss of vessel density appeared before cognitive impairment with no other gross brain pathology being present, suggesting cognitive impairment arose after brain capillary loss [44]. Damage to the subgranular zone of the hippocampal dentate gyrus also has been suggested as a mechanism of long term radiation induced cognitive impairment. Recent animal experiments have shown that this area is extremely sensitive to whole brain radiation [45]. Dosimetric planning for WBRT to spare the hippocampal region is already underway [46].

### Neuropsychological functioning of patients treated with radiation for brain metastases

For many patients with brain metastases, controlling neurological symptoms, preventing cognitive dysfunction, and maintaining functional independence are just as important as prolonging survival. Multiple factors, however, may negatively impact the neurocognitive functioning of these patients including the presence of the tumor, WBRT, SRS, neurosurgical procedures, chemotherapy, and other drugs that have neurotoxic effects such as steroids and anticonvulsants [47,48]. Research investigating the effects of treatment, including WBRT, on the neurocognitive functioning of patients with brain metastases is limited. While many studies have evaluated the neurocognitive outcome of patients treated with radiation, particularly children [49,50] and long term survivors of gliomas [51,52], the data from these populations are not directly comparable to patients undergoing WBRT and/or SRS for brain metastases. To examine the neurocognitive functioning of patients with brain metastases treated with radiation, some studies used the Folstein Mini-Mental State Examination (MMSE) [53] while more recent trials administered a battery of neuropsychological tests.

#### Neurocognitive impairments prior to radiation

Neurocognitive impairment in patients with brain metastases is common prior to receiving radiation treatment. In studies using the MMSE to assess neurocognitive status, 8 to 16% of patients were classified as having dementia [54-56] prior to receiving radiotherapy. Lower MMSE scores at baseline were associated with greater tumor volume [54,57] and death [55].

Neuropsychological testing was used in a phase III randomized trial to evaluate whether motexafin gadolinium administered with WBRT could improve neurologic and neurocognitive outcome and survival in patients with brain metastases [35,58]. This trial administered a brief battery of standardized neurocognitive tests assessing the domains of memory, executive function, and motor speed in 401 patients at study entry and at monthly intervals for the first six months and every three months until death [35,58]. Of these patients, 90.5% exhibited neurocognitive impairment prior to beginning WBRT, with 42% of the patients having impairment in at least four out of the eight tests administered. Similarly, another study using a neurocognitive test battery found that 67% of patients with one to three brain metastases were impaired on at least one test and 50% were impaired on two or more tests prior to radiation therapy [59]. In both of these trials, domains of functioning that tended to be the most impaired include fine motor dexterity, executive function, and memory, particularly immediate and delayed recall. The severity of neurocognitive impairment from brain lesions generally is related to the size of the tumor rather than the number of metastases. Meyers et al. [58] found that the volume of the indicator lesion was highly correlated with each neurocognitive test score at baseline. In addition, Chang et al. [59] found that patients with tumor volume greater than 3 cm^3 ^had worse performance on a measure of attention span.

Baseline neurocognitive function also is predictive of overall survival [58]. Tests of memory, motor dexterity, executive function, and global impairment were independent predictors of survival. When analyzed with other clinical parameters, impaired scores on the baseline Pegboard dominant hand test (a measure of fine motor dexterity) were found to be predictive of survival in addition to other factors such as male sex, number of brain metastases, and low KPS.

#### Neurocognitive function after WBRT

In the phase III randomized trial noted above, Meyers et al. [58] found that after treatment, overall neurocognitive test scores declined over time as patients progressed, with fine motor speed deteriorating the most (31% at 3 months) and verbal fluency the least (7% at 3 months). Changes in neurocognitive test scores correlated significantly with changes in tumor volume but not with the number of metastases. Patients with progressive disease showed greater deterioration in each neurocognitive function test compared to patients with partial response who demonstrated stable or improved performance on some tests. Furthermore, in a subset of patients with non-small-cell lung cancer, a prolonged time-to-neurocognitive progression for memory and executive function was found in patients treated with motexafin gadolinium and WBRT compared to WBRT alone, despite no difference between the two arms in overall survival or time to neurological or neurocognitive progression [58]. Thus, differential effects were found for specific neurocognitive functions supporting the use of neuropsychological testing in similar clinical trials.

Based on the 208 patients who received WBRT alone in the previously described phase III randomized trial [58], Li et al [60] investigated the relationship between tumor volume and neurocognitive function. Compared to poor responders, good responders exhibiting tumor reduction took longer to deteriorate in neurocognitive function on all tests but particularly on measures of executive function and fine motor speed. Similarly, tumor reduction correlated significantly with improvement in executive function and fine motor speed but not with changes in memory in a small sample of long-term survivors [60]. Thus, by reducing intracranial tumor burden, WBRT improves certain aspects of cognition. However, WBRT may have a specific negative effect on memory, which may be related to damage to the hippocampus. Patients surviving over one year had a greater reduction in tumor volume and better neurocognitive outcomes after WBRT than patients only surviving to four months [60]. A consistent finding from studies using either neuropsychological testing [58,60] or the MMSE [54,57] indicates that tumor control has a beneficial effect on neurocognitive function and quality of life.

In a secondary analysis of a study designed to test the feasibility of administering neuropsychological tests in brain metastasis patients [61], investigators looked at the short-term impact of WBRT on neurocognitive and quality of life measures [62]. They administered neuropsychological tests at baseline, the end of radiation therapy, and at one month follow up. Although declines in tests scores occurred immediately after radiation, improvements in neurocognitive and quality of life measures were found at one month post-WBRT compared to pre-WBRT, even in a group with limited expected survival. At one month follow up, the majority of patients exhibited improved or stable performance compared to baseline in memory, attention, and executive function. Li et al. [63], found that six months after WBRT, neurocognitive function predicted decline in QOL, as measured by activities of daily living, with Delayed Recall (memory) being the most predictive test. This finding suggests that delaying neurocognitive deterioration is important for preserving patients' quality of life. Since control of intracranial tumors, even for a short period of time, is associated with stabilization and improvement in neurocognitive function and quality of life, the use of WBRT outweigh the long-term risks in these patients [60].

#### Neurocognitive function after SRS

In a small pilot study evaluating neurocognitive function in patients receiving SRS alone for the treatment of one to three brain metastases [59], Chang et al. found that after one month all 13 patients declined on at least one neurocognitive test with about half showing decline on two or more tests. Patient's scores declined most frequently on tests of learning and memory (54%) and motor dexterity (46%). On other tests measuring executive function, attention, and verbal fluency, some patients exhibited improvements in their scores while others declined. Five patients were evaluated 200 days after their baseline evaluation to assess late cognitive effects. Stable or improved functioning was found in learning and memory in four patients and in executive function and motor dexterity in three patients. In this small study of long-term survivors of brain metastases treated with SRS alone, the majority demonstrated stable or improved neurocognitive functioning.

Aoyama et al. [57] used the MMSE to assess patients in their randomized trial evaluating SRS alone versus SRS plus WBRT. Their results showed that patients who received WBRT combined with SRS experienced a stable MMSE score for approximately 2 years after treatment compared with SRS alone. This is thought to be due to the preventative effect of WBRT on brain tumor recurrence.

Currently, there is an ongoing randomized Phase III clinical trial being run by the North Central Cancer Treatment Group (NCT00377156) and supported by the National Cancer Institute that does assess the neurocognitive effect of receiving either SRS alone or SRS followed by adjuvant WBRT in patients with three or fewer brain metastases. The trial's primary endpoint is overall survival but its secondary endpoints will evaluate quality of life and neurocognitive function by means of a battery of tests that evaluate memory, fluency, executive function, and coordination.

#### Improving neurocognitive function after WBRT

Multiple pharmacological agents have been proposed and are being investigated that could potentially improve cognition, mood, and quality of life in patients receiving radiation for brain tumors. These agents include methylphenidate, alpha-tocopherol, pentoxifylline and donepezil [64-67]. Currently there is an ongoing randomized phase III trial (RTOG 0614), testing memantine hydrochloride versus placebo in preventing cognitive dysfunction in patients undergoing WBRT for brain metastases. Mematine is a NMDA-receptor antagonist used in the treatment of Alzheimer disease. The study is using an extensive battery of neuropsychological assessments and quality of life measurements and is also collecting blood and urine specimens for future studies.

### The use of neuropsychological assessments

Neurocognitive function, which impacts quality of life [63,68], is an important outcome measure in clinical trials for cancer therapies. In some studies involving patients with brain metastases, the Folstein MMSE [53] has been used to assess neurocognitive function [54-57]. It is brief test that was designed to assess delirium or significant dementia. However, the MMSE does not adequately measure all the cognitive areas affected by radiation and is not a sensitive tool for detecting cognitive impairment in these patients [68,69] or changes related to therapeutic interventions [69]. Only 50% of patients having impaired cognitive function based on neuropsychological testing were considered abnormal on the MMSE [70]. Furthermore, scores on the MMSE did not change despite a decline in memory function assessed by neuropsychological testing. Thus, short batteries of objective standardized neuropsychological tests are recommended to assess cognitive functioning in clinical trials of patients with brain metastases.

Standardized neuropsychological tests are reliable and valid measures that are sensitive to changes in central nervous system function, and thus have been used as outcome measures in clinical trials. When selecting neuropsychological tests for use in clinical trials, several guidelines should be followed [71]. First, tests should be selected to assess the specific domains of functioning that may be affected by treatment. Second, tests need to be re-administered repeatedly, thus it is best to have alternate forms or tests that are more resistant to learning in order to minimize practice effects. Finally, the tests should be standardized measures with documented reliability and validity. In addition to these general criteria, several other considerations should be made when devising a test battery for use in clinical trials of patients with brain metastases [61]. First, these patients have a shortened lifespan and may feel fatigued, thus the test battery should be brief to facilitate compliance and lessen the burden on the patient. Second, the cost of the tests and level of staff training required to administer them should be considered, particularly for multi-center studies. Limited information is available regarding the appropriate neuropsychological tests to be used specifically in clinical trials for patients with brain metastases. However, there needs to be validation and consensus of an appropriate neuropsychological test battery for determining prognosis for treatment and for comparing the results of future clinical trials.

Recently, a phase III randomized trial used a brief battery of neuropsychological tests to generate more specific data about the neurocognitive effects of brain metastases before and after treatments [35,58,60,63]. These tests, which evaluate memory, verbal function, fine motor coordination, and executive function, provide a more accurate and comprehensive measurement of neuropsychological changes in patients with brain metastases who are treated with radiation therapy [68,69]. This short battery has a high compliance rate and can be completed in a reasonable time in patients with brain metastases [61,68]. To further develop neuropsychological testing for use in clinical trials of patients with brain metastases, the National Cancer Institute (NCI) Radiation Oncology Branch adapted the Meyers et al. [58,68] test battery by adding a few brief measures specifically to assess processing speed, working memory, and attention, which are functions that can be affected by radiation [47]. In addition, a test of estimated intelligence was included to serve as a measure of premorbid functioning. Besides these neuropsychological tests, measures of quality of life and activities of daily living also should be included in clinical trials to assess everyday functioning [72]. Examples of two such measures are the Barthel index and the Functional Assessment of Cancer Therapy-Brain (FACT-Br), which previously have been used in studies of patients with brain metastases[56,63]. The Barthel Index assesses daily living skills [73] and the FACT-Br was developed specifically to address the quality of life issues concerning brain tumor patients undergoing treatment [74]. Table 6 lists the measures that were compiled for an NCI research protocol that will examine which tests are sensitive to CNS changes in patients with brain metastases receiving WBRT. These tests are standardized, have alternate forms or are somewhat resistant to practice effects, and assess the main domains that may be affected by radiation. Furthermore, the battery takes less than one hour to administer, most of the tests are relatively inexpensive, and technicians can administer these tests appropriately when trained and supervised by a psychologist. The data generated from this protocol will be considered with findings from other studies to propose a standard neuropsychological test battery for use in the clinical trials of these patients to facilitate the comparison of different treatment regimens.

**Table 6:** Suggested neuropsychological test battery

**Psychometric Test**	**Domains**	**Time (mins)**
North American Adult Reading Test-35 [95]	Estimated Intelligence	5
Hopkins Verbal Learning Test [96] WAIS-III Digit Span subtest [97]	Memory	8 5
Ruff 2 & 7 Selective Attention Test [98]	Attention	5
WAIS-III Symbol Search subtest [97]	Processing Speed	2
Trail Making Test A & B [99] Controlled Oral Word Association Test [100]	Executive Function	5 5
Grooved Pegboard [101]	Motor Function	5
Barthel Index [73]	Adaptive Function	5
Functional Assessment of Cancer Therapy – Brain [74]	Quality of Life	5

	**Total Time**	**50 mins**

*Abbreviations: *WAIS-III = Wechsler Adult Intelligence Scale

Ultimately, having a brief test battery that is reliable and sensitive in detecting meaningful neuropsychological change in this patient population is very important. In the clinic, a condensed neuropsychological battery would be useful in monitoring cognitive and behavioral changes and predicting outcome. In research, a standardized neuropsychological test battery is an essential tool that needs to be incorporated into all future clinical trials investigating treatments for brain metastases. Such a battery should be used when assessing new radiation methods or delivery schemes and in trials investigating agents that modify radiation.

## Biomarkers as indicators of CNS injury

In addition to neuropsychological testing, biomarkers may be a useful research and prognostic tool. Elevated levels of certain proteins or neurotransmitters in the blood or urine may be indicators of CNS damage caused by invasion of brain metastases and/or radiation induced damage. Much of the work on biomarkers for CNS injury has been done in stroke patients. These studies have identified multiple markers of blood brain barrier disruption and neuronal damage. The various categories include markers of endothelial damage, excitotoxicity, inflammation and angiogenesis (Table 7).

**Table 7:** Biomarkers of CNS injury

Excitotoxicity	Glutamate GABA
	GABA
Endothelial Damage	Protein S100B
	NSE
	MMP-9
	MMP-13

Inflammation	TNF-alpha
	Il-1
	ICAM-1
	VCAM-1

Angiogenesis	MMP2
	MMP9
	VEGF

*Abbreviations: *NSE = neuron-specific enolase, MMP = matrix metalloproteinases

TNF = Tumor necrosis factor; Il = interleukin, ICAM = intercellular adhesion molecule; VCAM = vascular cellular adhesion molecule, VEGF = vascular endothelial growth factor

Two serum markers that have potential as screening tools for endothelial and neuronal damage are neuron-specific enolase (NSE) and S100B. NSE is a glycolytic enzyme found in the CNS, which is expressed by neural and neuroendocrine cells [75] and can be used as a marker of neuronal damage. Elevated levels have been found in patients with brain metastases from both small cell lung cancer and non-small cell lung cancer (NSCLC) [76]. A multi-center retrospective study involving 231 NSCLC patients demonstrated that high serum levels of NSE indicated shorter survival and was a specific marker of metastases [77].

S100B is a nervous system specific cytoplasmic protein found in astrocytes and is released into circulation when the blood brain barrier is breached [78]. It is elevated in stroke patients and its levels have been shown to correspond to infarct volume [79]. In a study looking at the presence of S100B in the serum of 38 patients with lung carcinoma, an elevated S100B level was either associated with brain metastases or with the presence of imaging changes suggestive of chronic, diffuse cerebral microvascular disease [80]. S-100 levels have also been shown to be a predictive marker of melanoma brain metastases [81].

Neuronal damage can lead to excitotoxicity where excess neurotransmitters such as glutamate and GABA are released. This increase in neurotransmitters causes an influx of Ca^2+ ^leading to Ca^2+ ^mediated cell death [82]. Excitotoxicity is seen in traumatic brain injury, ischemic stroke and neurodegenerative diseases. In addition, glutamate and GABA have been measured in the blood of patients who have had a stroke [83,84]. The release of neurotransmitters has never been studied in patients with brain metastasis or in patients with CNS damage caused by radiation but they also may be potential markers.

Radiation stimulates the inflammatory pathway and leads to the release of various cytokines, adhesion molecules and chemokines. Animal models have shown that radiation induced damage to the brain up regulates expression of TNF alpha, ICAM-1 and Il-1 [85]. These inflammatory markers already have been detected in the blood of patients who received radiation [86]. Radiation as well as CNS injury of any kind can cause release of these inflammation molecules. For example TNF alpha, ICAM and Il-1 all have been measured in the plasma of patients with stroke induced brain injury [87,88]. These never have been measured in patients receiving WBRT but they may be potential markers of CNS damage.

Angiogenic proteins released by metastatic cancer cells also may be used to monitor disease status and assist in predicting recurrence. Angiogenic factors have been investigated as possible tumor markers in various malignancies [89]. Vascular endothelial growth factor (VEGF) and matrix metalloproteinases (MMPs) have been shown to have prognostic value in various tumor types. A number of studies have demonstrated the role of VEGF and MMPs in breast [90], lung [91,92] and melanoma [93] metastases, but none specifically have examined blood or urine levels in patients with brain metastases. MMPs are not only involved in tumor invasion but can also be a sign of CNS vascular injury as indicated by an increase in plasma levels of MMP9 and MMP13 in stroke patients [94].

The NCI Radiation Oncology Branch protocol mentioned above that evaluates neuropsychological function also includes the collection of serum, plasma and urine specimens. The objective is to identify and evaluate the above biomarkers and to investigate the ability of these biomarkers to predict neuropsychological decline after WBRT and to predict progression of disease. The study will collect specimens before WBRT, at the completion of WBRT, and then at monthly intervals each coinciding with neuropsychological testing.

## Conclusion

WBRT is the standard of care in patients with brain metastases with surgery and SRS playing an important role when there are limited metastases. There are risks of neurocognitive impairment associated with WBRT; however omitting WBRT has been shown to be more detrimental in terms of survival and neurocognitive outcomes. It is also important to recognize that many patients present with neurocognitive deficits even before beginning radiotherapy. Many potential therapies being investigated also carry a risk of neurocognitive decline and the current focus of brain metastases research is to find ways to optimize the therapeutic index. Future clinical trials will be designed to answer questions such as the role of omitting upfront WBRT and giving SRS alone for a single metastasis, the benefit of administering prophylactic cranial irradiation to highly metastatic cancers such as HER2+ breast cancer patients, the value of using hippocampal sparing techniques, and the addition of radiosensitizers to enhance WBRT. To answer these questions and evaluate various treatment regimens that may have minimal differential effects on survival and disease progression, it is important to assess other patient outcomes [72], especially functions affected by neurotoxicity. Thus, tests of neuropsychological functioning should be included as standard outcome measures in all of these future studies. The challenge is finding a brief but sensitive and comprehensive test battery to assess the neurocognitive effects of brain metastases and treatments.

Biomarkers have potential in clinical research involving patients with brain metastases and are an avenue that needs to be explored. They may have diagnostic potential as well as potential for monitoring disease progression. Markers found in the blood may aid in understanding the pathophysiology of radiation induced CNS injury and assist in finding ways to target tumor cells while sparing healthy cells. In clinical trials involving radiomodifiers, biomarkers may be used to monitor the toxicity and effectiveness of these agents. Biomarkers may also have a role in predicting a decline in neurocognitive function. Ultimately, combining the outcomes of neuropsychological testing, biomarkers and imaging will help us improve the management of these patients.

## Competing interests

The authors declare that they have no competing interests.

## Authors' contributions

AB and KC participated in the conception of the work, compiled the information, reviewed and wrote the manuscript. PW participated in the conception of the work, development of the test battery, and review and writing of the manuscript.
